# Temporal Trends in Respiratory Infection Epidemics Among Pediatric Inpatients Throughout the Course of the COVID‐19 Pandemic From 2018 to 2023 in Fukushima Prefecture, Japan

**DOI:** 10.1111/irv.70070

**Published:** 2025-01-12

**Authors:** Yohei Kume, Koichi Hashimoto, Hisao Okabe, Sakurako Norito, Reiko Suwa, Miyuki Kawase, Izumi Mochizuki, Fumi Mashiyama, Naohisa Ishibashi, Shigeo Suzuki, Hiroko Sakuma, Kazuya Shirato, Mitsuaki Hosoya, Hayato Go

**Affiliations:** ^1^ Department of Pediatrics Fukushima Medical University Fukushima Japan; ^2^ Department of Virology III National Institute of Infectious Diseases Musashimurayama Tokyo Japan; ^3^ Department of Pediatrics Ohara General Hospital Fukushima Japan; ^4^ Department of Pediatrics Hoshi General Hospital Koriyama Fukushima Japan; ^5^ Department of Perinatology and Pediatrics for Regional Medical Support Fukushima Medical University Fukushima Japan

**Keywords:** child, COVID‐19, epidemiology, influenza viruses, metapneumovirus, respiratory syncytial viruses, SARS‐CoV‐2

## Abstract

**Background:**

Nonpharmaceutical interventions for coronavirus disease (COVID‐19), caused by severe acute respiratory syndrome coronavirus 2, during the pandemic altered the epidemiology of respiratory viruses. This study aimed to determine the changes in respiratory viruses among children hospitalized from 2018 to 2023.

**Methods:**

Nasopharyngeal specimens were collected from children aged under 15 years with fever and/or respiratory symptoms admitted to a medical institution in Fukushima Prefecture between January 2018 and December 2023. Eighteen respiratory viruses were detected using real‐time reverse transcription‐polymerase chain reaction.

**Results:**

Overall, 1933 patients were included. Viruses were detected in 1377 (71.2%); of these, a single virus was detected in 906 (46.9%) and multiple viruses in 471 (24.3%). Among the viruses whose epidemics were temporarily suppressed, the epidemics of respiratory syncytial virus A and human parainfluenza virus type 3 (HPIV3) started earlier, and the epidemics of human metapneumovirus, HPIV1, and influenza A and C viruses resumed as behavioral restrictions for preventing COVID‐19 eased. The median age of children with airway infection was significantly higher in the postpandemic group than in the prepandemic group (18.0 months vs. 21.0 months, *p* < 0.01). The median age of children infected with HPIV3 and human rhinovirus was significantly higher in the postpandemic group than in the prepandemic group.

**Conclusions:**

Strengthening of nonpharmaceutical interventions changed the epidemic dynamics of pediatric infectious diseases, with a trend toward older hospitalized children. Continuous monitoring of pediatric infectious disease outbreaks in hospitalized children can help prepare for the emergence of future viruses and pandemics.

## 1 Introduction

Understanding the dynamics of infectious disease epidemics is one of the most important issues in pediatric healthcare. Since severe acute respiratory syndrome coronavirus 2 (SARS‐CoV‐2) caused the first case of coronavirus disease (COVID‐19) in 2019 and the first case in Japan in January 2020 [1, 2], the epidemic dynamics of pediatric respiratory infections have changed significantly, assumedly as a result of the nonpharmaceutical interventions (NPIs), such as social distancing, travel restrictions, school closures, hand hygiene, and mask use [3], put in place to curb the pandemic. The thorough implementation of NPI against SARS‐CoV‐2 also temporarily controlled other respiratory virus infections [4, 5]. However, the number of rhinovirus cases did not markedly decrease [6, 7]. The suppression of infectious disease outbreaks may cause cumulative susceptibility of the population to viral infections, which may trigger subsequent outbreaks of respiratory diseases [8]. In May 2023, the Japanese government downgraded the status of COVID‐19 from Class 2 to Class 5, which includes common infectious diseases, such as seasonal influenza, to facilitate infection control [9].

The present study was conducted to clarify changes in the detection of respiratory viruses among hospitalized children in pediatric inpatient facilities in Fukushima Prefecture from the start of reinforcement of COVID‐19 prevention measures to the postrelaxation period and to better understand the epidemic dynamics of viral respiratory infections in children.

## 2 Methods

This study was conducted in accordance with the Declaration of Helsinki and approved by the Ethics Review Committee of Fukushima Medical University (No. 29006). Informed consent was obtained from the parents of the hospitalized children.

In this observational study, nasopharyngeal swab fluid was collected from all hospitalized children younger than 15 years of age with fever and/or respiratory symptoms (rhinorrhea, cough, wheezing) admitted to a single medical institution in Fukushima Prefecture between January 2018 and December 2023. We previously reported differences in respiratory virus epidemics before and after the COVID‐19 pandemic up to 2021 [7]. Subsequently, because of changes in the extent of social NPI for COVID‐19, we extended the study period to 2023. In addition, the present study also added patients who were hospitalized for fever alone to increase the number of viruses detected.

The samples were stored in a freezer at −80°C until use. Real‐time reverse transcription‐polymerase chain reaction (RT‐PCR) was used to detect the following 18 respiratory viruses: respiratory syncytial virus (RSV) A and B; influenza virus (Flu) A, B, and C; human coronavirus (HCoV) 229E, HKU1, NL63, and OC43; human metapneumovirus (HMPV); human parainfluenza (HPIV) 1, 2, 3, and 4; human rhinovirus (HRV); human adenovirus (HAdV) 2 and 4; and human bocavirus (HBoV). Detailed RT‐PCR methods, primer probes, and reaction conditions have been described in previous reports [7, 10, 11, 12]. Briefly, viral nucleic acids were extracted using the QIAamp Viral RNA Mini Kit (Qiagen, Hilden, Germany), QIAamp 96 Virus QIAcube HT Kit, or NucleoSpin 96 Virus kit (Macherey‐Nagel, Düren, Germany). One‐step RT‐PCR was performed using AgPath‐ID One‐Step RT‐PCR (Thermo Fisher Scientific, Waltham, MA, USA). Two‐step RT‐PCR was performed using LightCycler 480 Probes Master Mix (Roche, Basel, Switzerland). In the two‐step RT‐PCR, cDNA was generated using SMART MMLV Reverse Transcriptase (Takara Bio, Shiga, Japan) with Primer Random (Roche) and oligo (dT) primers (Qiagen).

Indications for hospitalization were determined by a pediatrician at the hospital. The primary reasons for hospitalization in children with fever and/or respiratory tract symptoms were decreased oxygen saturation requiring oxygen administration, poor oral intake, dehydration, poor overall health, and bacterial infections requiring antibiotics. Data on age, sex, and diagnosis at admission, including airway symptoms and blood test results (C‐reactive protein [CRP] and white blood cell counts), were obtained from the medical records.

In Japan, a state of emergency was declared for the first time in April 2020 to prevent the COVID‐19 pandemic. We categorized children hospitalized for respiratory tract infections before April 2020 as prepandemic and those hospitalized after April 2020 as postpandemic and statistically compared the ages of the hospitalized children. The Mann–Whitney *U* test was used to statistically compare continuous variables between groups. GraphPad Prism 9 (Graph Pad Software Inc., San Diego, CA, USA) was utilized for statistical analysis. *p* < 0.05 was considered as a statistically significant difference.

## 3 Results

In total, 1933 patients treated from 2018 to 2023 were included in the present study. The median age of the hospitalized patients was 20 months (interquartile range [IQR]: 10–42 months). Viruses were detected in 1377 cases (71.2%). Of these, 906 (46.9%) involved a single virus and 471 (24.3%) involved multiple viruses (Table 1).

**TABLE 1:** Characteristics of children hospitalized with respiratory infections in each year.

Years	2018–2023 (*n* = 1933)	2018 (*n* = 374)	2019 (*n* = 402)	2020 (*n* = 223)	2021 (*n* = 293)	2022 (*n* = 245)	2023 (*n* = 396)
	Median [IQR] /number (%)	Median [IQR] /number (%)	Median [IQR] /number (%)	Median [IQR] /number (%)	Median [IQR] /number (%)	Median [IQR] /number (%)	Median [IQR] /number (%)
Number of hospitalizations per month (/month)	24.3^a^	31.1^a^	33.5^a^	18.6^a^	24.4^a^	20.4^a^	33.0^a^
Age (months)	20 [10–42]	16 [8–30]	19 [10–42]	20 [12–58]	20 [10–36]	21 [10–37]	24 [11–54]
Sex (male)	1065 (55.1)	199 (53.2)	226 (56.2)	126 (56.5)	155 (52.9)	131 (53.5)	228 (57.6)
Virus detection	1377 (71.2)	282 (75.4)	304 (75.6)	137 (61.4)	210 (71.7)	164 (66.9)	280 (70.7)
Single virus detection	906 (46.9)	165 (44.1)	185 (46.0)	105 (47.1)	152 (51.9)	108 (44.1)	191 (48.2)
Multiple virus detection	471 (24.3)	117 (31.2)	119 (29.6)	32 (14.3)	58 (19.7)	56 (22.8)	89 (22.4)
RSV A	307 (15.8)	43 (11.1)	77 (19.2)	10 (4.5)	97 (33.1)	42 (17.1)	38 (9.6)
RSV B	234 (12.1)	82 (21.9)	75 (18.7)	3 (1.3)	9 (3.1)	0 (0.0)	65 (16.4)
Flu A	49 (2.5)	9 (2.4)	15 (3.7)	10 (4.5)	0 (0.0)	0 (0.0)	15 (3.8)
Flu B	10 (0.5)	6 (1.6)	3 (0.7)	1 (0.4)	0 (0.0)	0 (0.0)	0 (0.0)
Flu C	21 (1.1)	5 (1.3)	0 (0.0)	0 (0.0)	0 (0.0)	0 (0.0)	16 (4.0)
HCoV 229E	12 (0.6)	1 (0.3)	8 (62.0)	3 (1.3)	0 (0.0)	1 (0.4)	0 (0.0)
HCoV OC43	41 (2.1)	6 (1.6)	4 (1.0)	8 (3.6)	13 (4.4)	0 (0.0)	10 (2.5)
HCoV NL63	27 (1.4)	3 (0.8)	7 (1.7)	0 (0.0)	12 (4.1)	1 (0.4)	4 (1.0)
HCoV HKU1	27 (1.4)	4 (1.1)	5 (1.2)	6 (2.7)	0 (0.0)	0 (0.0)	12 (3.0)
HMPV	148 (7.7)	37 (9.9)	37 (9.2)	23 (10.3)	0 (0.0)	22 (9.0)	29 (7.3)
HPIV 1	36 (1.9)	9 (2.4)	5 (1.2)	1 (0.4)	0 (0.0)	14 (5.7)	7 (1.8)
HPIV 2	17 (0.9)	0 (0.0)	5 (1.2)	0 (0.0)	1 (0.3)	2 (0.8)	9 (2.3)
HPIV 3	107 (5.5)	28 (7.5)	28 (7.0)	1 (0.4)	20 (6.8)	1 (0.4)	29 (7.3)
HPIV 4	36 (1.9)	4 (1.1)	10 (2.5)	1 (0.4)	6 (2.0)	4 (1.6)	11 (2.8)
HAdV 2	215 (11.1)	59 (15.8)	39 (9.7)	23 (10.3)	23 (7.8)	38 (15.5)	33 (8.3)
HAdV 4	39 (3.1)	13 (3.5)	26 (6.5)	7 (3.1)	4 (1.4)	3 (1.2)	6 (1.5)
HBoV	236 (12.2)	68 (18.2)	50 (12.4)	25 (11.2)	41 (14.0)	30 (12.2)	22 (5.6)
HRV	370 (19.1)	52 (13.9)	67 (16.7)	54 (24.2)	49 (16.7)	67 (27.3)	81 (20.5)

*Note:* Data are presented as median (IQR) or numerical values (%).

Abbreviations: Flu, influenza virus; HAdV, human adenovirus; HBoV, human bocavirus; HCoV, human coronavirus; HMPV, human metapneumovirus; HPIV, human parainfluenza virus; HRV, human rhinovirus; IQR, interquartile range; RSV, respiratory syncytial virus.

^a^ Number of children hospitalized for fever and/or respiratory symptoms in the month.

**FIGURE 1:**
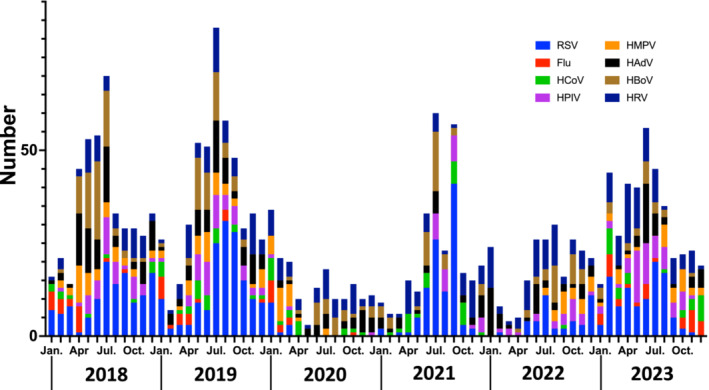
Successive changes in respiratory viruses detected using real‐time RT‐PCR from 2018 to 2023. Flu, influenza virus; HAdV, human adenovirus; HBoV, human bocavirus; HCoV, human coronavirus; HMPV, human metapneumovirus; HPIV, human parainfluenza virus; HRV, human rhinovirus; RSV, respiratory syncytial virus; RT‐PCR, reverse transcription‐polymerase chain reaction.

Among the 6 years of the study period, the fewest children were hospitalized in 2020, when the pandemic began (18.6 cases/month). By 2023, the number of hospitalized children had recovered to the prepandemic level (33.0 cases/month). The virus detection rate (61.4%, 137/223) and virus duplicate detection rate (14.3%, 32/223) in 2020 were lower than those in other years; however, by 2023, these rates had increased to 70.7% (280/396) and 22.4% (89/396), respectively. The median ages of hospitalized children in 2022 (median: 21 months, IQR: 10–37 months) and 2023 (median: 24 months, IQR: 11–54 months) showed an upward trend, compared with 2018 (median: 16 months, IQR: 8–30 months) and 2019 (median: 19 months, IQR: 10–42 months) (Table 1). Figure 1 shows the changes in the total number of viruses detected during the study period. In cases in which multiple viruses were detected in a single sample, each virus was counted. The detection of the virus decreased after April 2020, when Japan first declared an emergency state for the COVID‐19 pandemic; however, the number of viruses detected has been increasing again since June 2021. The number of virus detections increased in summer each year, except in 2020.

**FIGURE 2:**
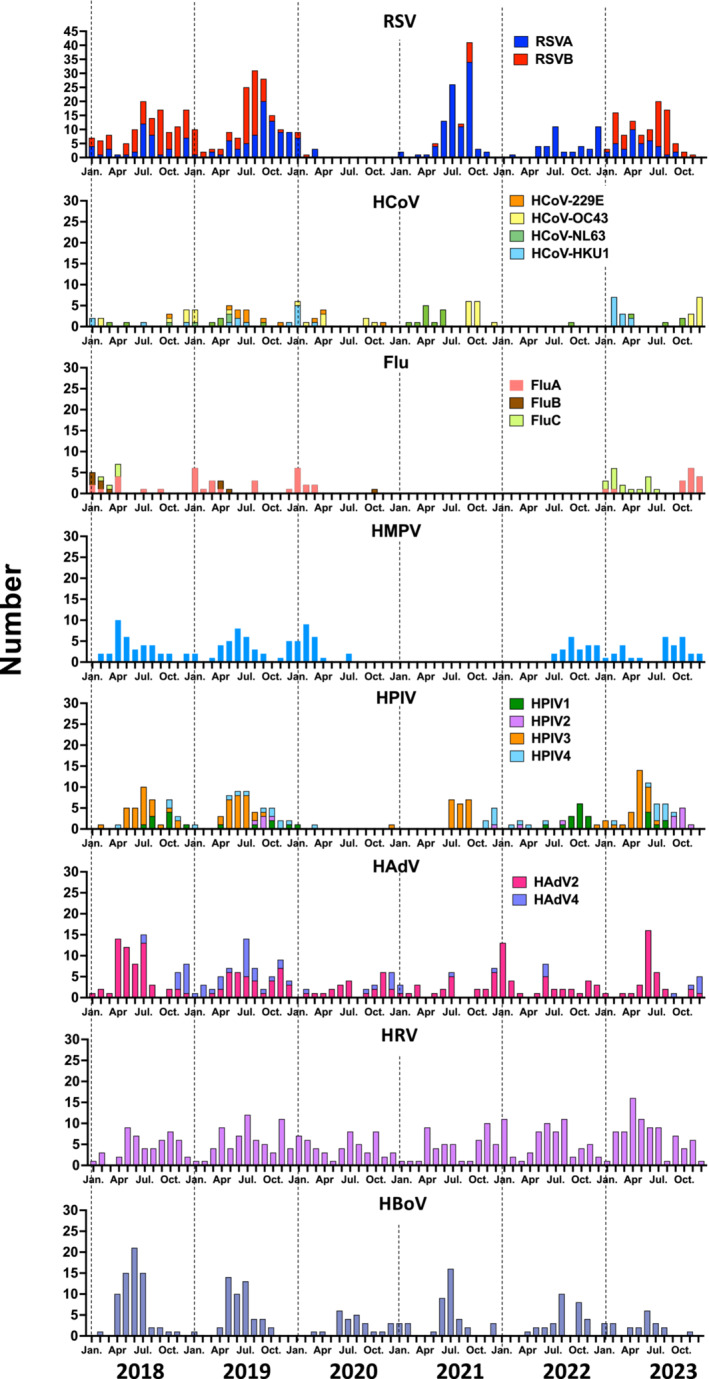
Changes in the number of detections of each virus during the study period from 2018 to 2023. Flu, influenza virus; HAdV, human adenovirus; HBoV, human bocavirus; HCoV, human coronavirus; HMPV, human metapneumovirus; HPIV, human parainfluenza virus; HRV, human rhinovirus; RSV, respiratory syncytial virus.

Changes in the epidemic dynamics of the 18 respiratory viruses detected from January 2018 to December 2023 are shown in Figure 2. Certain numbers of HRV, HAdV, and HBoV were detected after the COVID‐19 pandemic, although the number of detected cases has decreased. RSV, HPIV, HMPV, and Flu were virtually undetectable starting in April 2020. The number of hospital admissions owing to RSV and HPIV infections increased again in the summer of 2021. There has been an increase in the number of HMPV detections in hospitalized patients since July 2022, whereas the number of cases in which Flu was detected has increased since 2023. In particular, the number of detections of Flu C increased in early 2023, whereas the number of detections of Flu A increased after fall. In contrast, Flu B and HCoV‐229E were barely detectable from the start of the COVID‐19 pandemic until 2023.

The clinical characteristics of each virus are shown in Table 2. The median age of the hospitalized children was the highest in Flu cases (median: 43 months, IQR: 17–77 months) and lowest in RSV cases (median: 15 months, IQR: 4–28 months). RSV and HMPV infections were associated with higher rates of coughing, whereas RSV, HCoV, and HRV infections were associated with higher rates of wheezing. The rate of duplicate viral detection was higher for HBoV. The Flu group had the shortest time from symptom onset to hospitalization (median: 2.0 days; IQR: 1.0–4.0 days). The CRP level at admission was the highest in cases of HAdV (median: 2.1 mg/dL; IQR: 0.8–4.6 mg/dL).

**TABLE 2:** Clinical characteristics of each virus‐infected hospitalized child.

	RSV (*n* = 541)	Flu (*n* = 80)	HCoV (*n* = 104)	HMPV (*n* = 148)	HPIV (*n* = 190)	HAdV (*n* = 269)	HBoV (*n* = 236)	HRV (*n* = 370)
	Median [IQR] /number (%)	Median [IQR] /number (%)	Median [IQR] /number (%)	Median [IQR] /number (%)	Median [IQR] /number (%)	Median [IQR] /number (%)	Median [IQR] /number (%)	Median [IQR] /number (%)
Age (months)	15 [4–28]	43 [17–77]	25 [14–43]	22 [13–39]	19 [12–39]	19 [12–35]	17 [11–27]	18 [9–36]
Sex (male)	281 (51.9)	42 (52.5)	59 (55.7)	80 (54.1)	107 (56.3)	159 (59.1)	120 (50.8)	209 (56.5)
Fever	462 (85.4)	77 (96.3)	95 (91.3)	139 (93.9)	183 (96.3)	251 (93.3)	219 (92.8)	319 (86.2)
Rhinorrhea	417 (77.1)	41 (51.3)	62 (59.6)	113 (76.4)	122 (64.2)	175 (65.1)	150 (62.6)	237 (64.1)
Cough	492 (90.9)	58 (72.5)	75 (72.1)	133 (89.9)	144 (75.8)	185 (68.8)	165 (69.9)	261 (70.5)
Wheezing	136 (25.1)	8 (10.0)	22 (21.2)	20 (13.5)	25 (13.2)	32 (11.9)	40 (16.9)	94 (25.4)
Single virus detection	323 (59.7)	49 (61.2)	40 (38.5)	78 (52.7)	84 (47.3)	92 (34.2)	63 (26.7)	176 (47.6)
Multiple virus detection	218 (40.3)	31 (38.8)	64 (61.5)	70 (47.3)	106 (55.7)	177 (65.8)	173 (73.3)	194 (52.4)
Period from onset to Hospitalization (days)	4.0 [2.0–5.0]	2.0 [1.0–4.0]	3.0 [2.0–4.3]	4.0 [3.0–5.0]	4.0 [2.0–5.0]	3.0 [2.0–5.0]	4.0 [2.0–5.0]	3.0 [1.0–4.0]
Hospitalization period (days)	5.0 [4.0–6.0]	3.0 [3.0–5.0]	5.0 [4.0–6.0]	5.0 [4.0–6.0]	5.0 [4.0–6.0]	5.0 [4.0–6.0]	5.0 [4.0–6.0]	5.0 [4.0–6.0]
WBC (cells/μL)	9500 [7200–12,600]	8200 [5750–12,350]	10,400 [7375–15,025]	8900 [6600–11,575]	9500 [6900–13,200]	12,200 [9100–16,400]	10,600 [7700–14,525]	12,050 [9125–15,400]
CRP (mg/dL)	0.9 [0.2–2.4]	1.1 [0.3–2.6]	1.1 [0.3–3.1]	1.6 [0.7–3.3]	1.3 [0.3–4.8]	2.1 [0.8–4.6]	1.8 [0.5–4.1]	1.4 [0.4–4.1]

Abbreviations: CRP, C‐reactive protein; Flu, influenza virus; HAdV, human adenovirus; HBoV, human bocavirus; HCoV, human coronavirus; HMPV, human metapneumovirus; HPIV, human parainfluenza virus; HRV, human rhinovirus; IQR, interquartile range; RSV, respiratory syncytial virus; WBC, white blood cell.

Table 3 shows a comparison of the age of hospitalized children with respiratory tract infections before and after COVID‐19. The median age of children with airway infection was significantly higher in the postpandemic group than that in the prepandemic group (18.0 months vs. 21.0 months, *p* < 0.01). The median age of children infected with HPIV 3 and HRV was significantly higher in the postpandemic group than in the prepandemic group. Hospitalized children infected with Flu C tended to be older in the postpandemic group than in the prepandemic group, although the differences were not statistically significant (12.0 months vs. 19.0 months, *p* = 0.05). Furthermore, the trends in the age of children hospitalized due to each viral infection between 2018 and 2023 are shown in Table S1.

**TABLE 3:** Comparison of ages of hospitalized children with respiratory tract infections before and after the COVID‐19 pandemic.

	Prepandemic		Postpandemic		*p*
	*N*	Median (IQR)	*N*	Median (IQR)	
Total	853	18.0 (9.0–39.9)	1080	21.0 (11.0–44.0)	**< 0.01**
RSV A	130	15.0 (5.0–28.0)	177	17.0 (4.0–31.8)	0.68
RSV B	160	12.0 (4.0–22.0)	74	14.0 (4.0–28.0)	0.17
Flu A	34	46.5 (20.3–77.5)	15	67.0 (42.5–91.5)	0.15
Flu C	5	12.0 (10.0–16.0)	16	19.0 (15.0–44.5)	0.05
HCoV OC43	12	20.5 (7.8–37.5)	29	22.0 (13.0–43.0)	0.33
HCoV NL63	10	23.0 (17.3–32.5)	17	23.0 (13.0–27.0)	0.80
HCoV HKU1	15	34.0 (19.0–46.5)	12	42.0 (21.5–52.8)	0.56
HMPV	94	21.0 (13.0–36.0)	54	22.5 (12.0–44.3)	0.52
HPIV 1	15	27.0 (10.5–43.5)	21	26.0 (14.0–38.0)	0.84
HPIV 2	5	66.0 (12.0–74.0)	12	63.5 (29.8–78.8)	0.72
HPIV 3	56	13.0 (9.8–21.0)	51	22.0 (13.5–40.5)	**< 0.01**
HPIV 4	15	16.0 (11.5–23.0)	21	19.0 (14.0–50.0)	0.18
HAdV 2	100	17.5 (11.0–28.0)	115	19.0 (12.3–31.0)	0.44
HAdV 4	40	29.5 (11.8–47.0)	19	19.0 (16.0–40.0)	0.88
HBoV	119	17.0 (11.5–27.0)	117	16.0 (11.0–26.0)	0.97
HRV	136	14.0 (6.0–28.0)	234	19.0 (11.0–37.0)	**< 0.01**

*Note:* Age is shown in months. Mann–Whitney's U test was used to statistically compare data between the two groups.

Abbreviations: COVID‐19, coronavirus disease 2019; Flu, influenza virus; HAdV, human adenovirus; HBoV, human bocavirus; HCoV, human coronavirus; HMPV, human metapneumovirus; HPIV, human parainfluenza virus; HRV, human rhinovirus; IQR, interquartile range; RSV, respiratory syncytial virus.

## 4 Discussion

This study revealed the epidemic status of pediatric respiratory infections following the COVID‐19 pandemic. HRV, HBoV, and HAdV2 have been detected in certain numbers even after the COVID‐19 pandemic. The number of virus detections and duplicate detection rates were the lowest in 2020 but then increased from 2021 onward. Furthermore, the age of hospitalized patients increased after the COVID‐19 pandemic.

In the aftermath of the COVID‐19 pandemic, public health measures, such as hand hygiene and avoiding human‐to‐human contact, have reduced the prevalence of respiratory viral infections and other infectious diseases. In addition to reports of droplet infections, reports of diseases transmitted through the fecal‐oral route (rotavirus, norovirus, HAdV, and hepatitis A) and contact infections (epidemic keratoconjunctivitis) have decreased [13, 14]. Meanwhile, the COVID‐19 pandemic did not significantly affect the number of sexually transmitted infections (syphilis and acquired immunodeficiency syndrome), exanthema subitum caused by household infections, or carbapenem‐resistant *Enterobacteriaceae*, which are mostly nosocomial infections [14]. As for respiratory tract infections, the enveloped viruses—RSV, HMPV, and Flu—were rarely detected in hospitalized children immediately after the COVID‐19 pandemic, whereas the nonenveloped viruses—HRV and HAdV—were not completely suppressed [6, 7, 15]. These findings may be caused by the inability to completely inactivate nonenveloped viruses via hand disinfection with alcohol or soap.

Compared with the pre–COVID‐19 pandemic period, the postpandemic period saw a rise in the median age of hospitalized children, especially those with HRV, HPIV3, and Flu C infections. Previous studies have demonstrated an increase in the age in months of hospitalized children infected with RSV after the COVID‐19 pandemic [16]. In Denmark, an increase in hospitalizations and ventilator use was reported among older RSV‐infected children [17]. Furthermore, an increase in the age of children hospitalized not only for RSV but also for hMPV was reported [18]. This increase can be attributed to the increased rate of primary transmission among relatively older children as a result of their susceptibility to respiratory tract infections during the pandemic, indicating the need for regular exposure to the virus to maintain immunity and prevent the hospitalization of children.

In the present study, the number of children hospitalized with Flu C infection increased in the winter and spring of 2023. Flu C infections are associated with fewer days of fever, milder illness, and fewer hospitalizations than those of Flu A and B, and clinical symptoms are rarely a problem in cases of reinfection with Flu C [19]. Flu C–related hospitalizations are most common in children under 3 years of age. Seasonality is generally reported to be prevalent in winter and spring, although there have been reports of epidemics in summer [19]. Regarding the epidemic cycle of Flu C, in Japan, epidemics were reported to occur in even‐numbered years [20], whereas a French study showed that no epidemic was observed for 2 years after the peak of influenza C detection in 2005 [21]. There is no consensus on the seasonality or epidemic cycle of Flu C; possibly, the 2023 hospitalizations due to Flu C infection occurred during a Flu C epidemic cycle. Population immunity to Flu C may have declined at least after the implementation of NPI during the COVID‐19 pandemic, which may have contributed to an increase in the number of hospitalizations for Flu C and in the age of hospitalized children in months.

The median age in months of children infected with 11 of the 16 viruses, excluding the two viruses (Flu B and HCoV‐229E) that were not prevalent after the pandemic, had increased in the present study. The age in months of children hospitalized for RSV and hMPV infections also showed an increase before and after the COVID‐19 pandemic, although the increase in age was not significant. However, apropos the age of affected children at which each virus was detected over time, the median age increased after 2021 for RSV A, and the median age of affected children was the highest in 2022 for HMPV, when the epidemic resurfaced (Table S1). In view of these results, the results of the present study can be considered consistent with those of previous studies. After the COVID‐19 pandemic, HPIV 3 epidemics occurred in 2021 and 2023, and its suppression in 2020 and 2022 (Table S1). Each of these two epidemics and their suppression may have contributed to the resultant increase in the age of affected children hospitalized for HPIV 3 infection. Meanwhile, it is not clear why the age of HRV‐infected children increased after 2020; it may be that the NPI suppressed the prevalence of many enveloped viruses, thereby weakening other viral interferences with HRV and spreading the infection across a wide age range. Nevertheless, factors that influenced the age structure of HRV‐infected children remain unknown and require further investigation.

Depending on the conditions of the patients under study and the method of virus detection, previous reports have shown a virus detection rate of approximately 48%–85% for respiratory tract infections [22, 23, 24, 25, 26] and a virus duplication detection rate of approximately 16%–37% [22, 23, 26]. Duplicate detection is not necessarily the result of pathogenic viruses that contribute to pathogenesis. A previous report indicated that one respiratory virus can prevent infection by another by stimulating antiviral defenses in the airway mucosa [27]. In contrast, multiple viruses can grow simultaneously using air‐liquid interface cultures of human bronchial/tracheal epithelial cells from specimens of children with airway symptoms in which two or more viruses were detected by real‐time PCR [28]. Future studies are needed to evaluate the clinical significance of the duplication of viral infections.

RSVA, HPIV3, HCoV‐OC43, and HCoV‐NL63 were epidemic in Japan before the NPI for SARS‐CoV‐2 was removed, whereas HMPV and influenza A were epidemic after the NPI was removed. The factors that determined the sequence of respiratory virus epidemics resuming are unclear. However, the factors contributing to the earlier epidemic of RSV include the fact that RSV causes bronchiolitis, which is a more prominent cause of hospitalization in young children than other enveloped viruses [29], RSV has a higher basic reproduction number than other respiratory viruses [30], and RSV infections occur more frequently in daycare facilities and families with young children. In addition, enforcing NPI among young children, such as wearing masks, proved difficult even during the COVID‐19 pandemic, and the combination of conditions for the spread of RSV infection, such as loosening of NPI due to habituation, may have contributed to the relatively early resumption of the RSV outbreak. Although the details of HPIV3 and HCoVs spread are not known, its epidemic is thought to have been induced by the same conditions as those reported for RSV.

This study has some limitations. First, it was conducted at a single facility. However, this medical facility is the core hospital for pediatric care in the Fukushima Prefecture, and these results should accurately reflect the epidemic status of pediatric respiratory infections. Second, the study does not account for potential confounders such as variations in NPIs across different regions. Third, viral infections that cause relatively weak symptoms often do not result in hospitalization, leading to an underestimation of the actual number of epidemics in the area. However, the key issue with pediatric infections is whether they require hospitalization. This study focused on viruses detected in hospitalized children to identify viral infections that should be noted in this population.

Our findings revealed the epidemic dynamics of pediatric viral infections during the COVID‐19 pandemic. Among the viruses whose epidemics were temporarily suppressed, the epidemics of RSVA and HPIV3 started earlier than those of other viruses, and the epidemics of HMPV, HPIV1, Flu A and C, and other viruses resumed as behavioral restrictions gradually eased. The changing social conditions associated with the pandemic have also affected other respiratory viral infections, thereby changing the epidemic dynamics of various viruses. To prepare for the emergence of new viruses and future pandemics, it is important to continue monitoring pediatric infectious disease outbreaks among hospitalized children.

## Author Contributions

**Yohei Kume:** conceptualization, data curation, formal analysis, writing – original draft, methodology, software. **Koichi Hashimoto:** conceptualization, supervision, writing – review and editing, methodology. **Hisao Okabe:** investigation, data curation. **Sakurako Norito:** data curation, investigation. **Reiko Suwa:** investigation. **Miyuki Kawase:** investigation. **Izumi Mochizuki:** investigation. **Fumi Mashiyama:** investigation. **Naohisa Ishibashi:** investigation. **Shigeo Suzuki:** investigation. **Hiroko Sakuma:** investigation. **Kazuya Shirato:** investigation, supervision, methodology. **Mitsuaki Hosoya:** supervision. **Hayato Go:** supervision.

## Ethics Statement

This study was conducted in accordance with the Declaration of Helsinki and approved by the Ethics Review Committee of Fukushima Medical University (No. 29006).

## Consent

Informed consent was obtained from the parents of the hospitalized children.

## Conflicts of Interest

The authors declare no conflicts of interest.

### Peer Review

The peer review history for this article is available at https://www.webofscience.com/api/gateway/wos/peer‐review/10.1111/irv.70070.

## Supporting information


**Table S1.** Trends in the age of hospitalized children according to the virus from 2018 to 2023

## Data Availability

The data that support the findings of this study are available from the corresponding author upon reasonable request.

## References

[1] K. Shirato , N. Nao , H. Katano , et al., “Development of Genetic Diagnostic Methods for Detection for Novel Coronavirus 2019 (nCoV‐2019) in Japan,” Japanese Journal of Infectious Diseases 73 (2020): 304–307. 32074516 10.7883/yoken.JJID.2020.061

[2] N. Nao , K. Shirato , S. Matsuyama , and M. Takeda , “Detection of WN‐Human1 Sequence from Clinical Specimen,” accessed August 27, 2024, https://www.niid.go.jp/niid/images/vir3/nCoV/Method_NIID_20200114_Na.pdf.

[3] R. E. Baker , S. W. Park , W. Yang , G. A. Vecchi , C. Jessica , and B. T. Grenfell , “The Impact of COVID‐19 Nonpharmaceutical Interventions on the Future Dynamics of Endemic Infections,” Proceedings of the National Academy of Sciences of the United States of America 117 (2020): 30547–30553. 33168723 10.1073/pnas.2013182117 PMC7720203

[4] H. J. Shi , N. Y. Kim , S. A. Eom , et al., “Effects of Non‐Pharmacological Interventions on Respiratory Viruses Other Than SARS‐CoV‐2: Analysis of Laboratory Surveillance and Literature Review From 2018 to 2021,” Journal of Korean Medical Science 37 (2022): 1–13. 10.3346/jkms.2022.37.e172 PMC9151990 35638198

[5] N. Terliesner , N. Unterwalder , A. Edelmann , et al., “Viral Infections in Hospitalized Children in Germany During the COVID‐19 Pandemic: Association With Non‐Pharmaceutical Interventions,” Frontiers in Pediatrics 10 (2022): 935483. 36034546 10.3389/fped.2022.935483 PMC9403271

[6] E. Takashita , C. Kawakami , T. Momoki , et al., “Increased Risk of Rhinovirus Infection in Children During the Coronavirus Disease‐19 Pandemic,” Influenza and Other Respiratory Viruses 15 (2021): 488–494. 33715290 10.1111/irv.12854 PMC8189209

[7] Y. Kume , K. Hashimoto , M. Chishiki , et al., “Changes in Virus Detection in Hospitalized Children Before and After the Severe Acute Respiratory Syndrome Coronavirus 2 Pandemic,” Influenza and Other Respiratory Viruses 16 (2022): 837–841. 35488324 10.1111/irv.12995 PMC9343337

[8] E. J. Chow , T. M. Uyeki , and H. Y. Chu , “The Effects of the COVID‐19 Pandemic on Community Respiratory Virus Activity,” Nature Reviews. Microbiology 21 (2023): 195–210. 36253478 10.1038/s41579-022-00807-9 PMC9574826

[9] Ministry of Health Labour and Welfare , Response to COVID‐19 (Novel Coronavirus) After the Classification Change,” 2023, Accessed August 27, 2024, https://www.mhlw.go.jp/stf/covid‐19/kenkou‐iryousoudan_00006.html.

[10] K. Shirato , R. Suwa , N. Nao , et al., “Molecular Epidemiology of Human Metapneumovirus in East Japan Before and After COVID‐19, 2017‐2022,” Japanese Journal of Infectious Diseases 77 (2024): 137–143. 38171847 10.7883/yoken.JJID.2023.350

[11] Y. Kume , K. Hashimoto , K. Shirato , et al., “Epidemiological and Clinical Characteristics of Infections With Seasonal Human Coronavirus and Respiratory Syncytial Virus in Hospitalized Children Immediately Before the Coronavirus Disease 2019 Pandemic,” Journal of Infection and Chemotherapy 28 (2022): 859–865. 35307263 10.1016/j.jiac.2022.03.001 PMC8920880

[12] R. Suwa , Y. Kume , M. Kawase , et al., “Practical Validation of United States Centers for Disease Control and Prevention Assays for the Detection of Human Respiratory Syncytial Virus in Pediatric Inpatients in Japan,” Pathogens 11 (2022): 754. 35889999 10.3390/pathogens11070754 PMC9319774

[13] Y. Fukuda , T. Tsugawa , Y. Nagaoka , et al., “Surveillance in Hospitalized Children With Infectious Diseases in Japan: Pre‐ and Post‐Coronavirus Disease 2019,” Journal of Infection and Chemotherapy 27 (2021): 1639–1647. 34389224 10.1016/j.jiac.2021.07.024 PMC8332734

[14] K. Hibiya , H. Iwata , T. Kinjo , et al., “Incidence of Common Infectious Diseases in Japan During the COVID‐19 Pandemic,” PLoS ONE 17 (2022): 1–22. 10.1371/journal.pone.0261332 PMC8754328 35020724

[15] H. M. Kim , E. J. Lee , N. J. Lee , et al., “Impact of Coronavirus Disease 2019 on Respiratory Surveillance and Explanation of High Detection Rate of Human Rhinovirus During the Pandemic in the Republic of Korea,” Influenza and Other Respiratory Viruses 15 (2021): 721–731. 34405546 10.1111/irv.12894 PMC8446939

[16] D. A. Foley , D. K. Yeoh , C. A. Minney‐Smith , et al., “The Interseasonal Resurgence of Respiratory Syncytial Virus in Australian Children Following the Reduction of Coronavirus Disease 2019‐Related Public Health Measures,” Clinical Infectious Diseases 73 (2021): e2829–e2830. 33594407 10.1093/cid/ciaa1906 PMC7929151

[17] U. Nygaard , U. B. Hartling , J. Nielsen , et al., “Hospital Admissions and Need for Mechanical Ventilation in Children With Respiratory Syncytial Virus Before and During the COVID‐19 Pandemic: A Danish Nationwide Cohort Study,” Lancet Child & Adolescent Health 7 (2023): 171–179. 36634692 10.1016/S2352-4642(22)00371-6 PMC9940917

[18] M. Nagasawa , T. Udagawa , M. Okada , et al., “COVID‐19 Pandemic‐Altered Epidemiology of Respiratory Syncytial Virus and Human Metapneumovirus Infections in Young Children,” GHM Open 4 (2024): 47–49. 10.35772/ghmo.2024.01001 PMC11933982 40144746

[19] B. K. Sederdahl and J. V. Williams , “Epidemiology and Clinical Characteristics of Influenza C Virus,” Viruses 12 (2020): 12. 10.3390/v12010089 PMC7019359 31941041

[20] Y. Matsuzaki , K. Sugawara , C. Abiko , et al., “Epidemiological Information Regarding the Periodic Epidemics of Influenza C Virus in Japan (1996–2013) and the Seroprevalence of Antibodies to Different Antigenic Groups,” Journal of Clinical Virology 61 (2014): 87–93. 25017953 10.1016/j.jcv.2014.06.017

[21] S. Gouarin , A. Vabret , J. Dina , et al., “Study of Influenza C Virus Infection in France,” Journal of Medical Virology 80 (2008): 1441–1446. 18551600 10.1002/jmv.21218 PMC7166557

[22] A. Kaida , H. Kubo , K. Takakura , et al., “Associations Between Co‐Detected Respiratory Viruses in Children With Acute Respiratory Infections,” Japanese Journal of Infectious Diseases 67 (2014): 469–475. 25410563 10.7883/yoken.67.469

[23] J. Tan , J. Wu , W. Jiang , et al., “Etiology, Clinical Characteristics and Coinfection Status of Bronchiolitis in Suzhou,” BMC Infectious Diseases 21 (2021): 135. 33522910 10.1186/s12879-021-05772-x PMC7851904

[24] Y. Zhang , L. Su , Y. Chen , et al., “Etiology and Clinical Characteristics of SARS‐CoV‐2 and Other Human Coronaviruses Among Children in Zhejiang Province, China 2017‐2019,” Virology Journal 18 (2021): 89. 33931105 10.1186/s12985-021-01562-8 PMC8085659

[25] Z. Haddadin , J. Chappell , R. McHenry , et al., “Coronavirus Surveillance in a Pediatric Population in Jordan From 2010 to 2013: A Prospective Viral Surveillance Study,” Pediatric Infectious Disease Journal 40 (2021): e12–e17. 33165274 10.1097/INF.0000000000002965

[26] C. Calvo , S. Alcolea , I. Casas , et al., “A 14‐Year Prospective Study of Human Coronavirus Infections in Hospitalized Children: Comparison With Other Respiratory Viruses,” Pediatric Infectious Disease Journal 39 (2020): 653–657. 32453196 10.1097/INF.0000000000002760

[27] A. Wu , V. T. Mihaylova , M. L. Landry , and E. F. Foxman , “Interference Between Rhinovirus and Influenza A Virus: A Clinical Data Analysis and Experimental Infection Study,” Lancet Microbe 1 (2020): e254–e262. 33103132 10.1016/s2666-5247(20)30114-2 PMC7580833

[28] M. Kawase , R. Suwa , S. Sugimoto , et al., “Evidence of the Simultaneous Replications of Active Viruses in Specimens Positive for Multiple Respiratory Viruses,” Microbiology Spectrum 12 (2024): e0192023. 38051050 10.1128/spectrum.01920-23 PMC10783086

[29] R. Article , “More on Viral Bronchiolitis in Children,” New England Journal of Medicine 375 (2016): 1199–1200. 10.1056/NEJMc1607283 27653587

[30] J. A. Spencer , D. P. Shutt , S. K. Moser , et al., “Distinguishing Viruses Responsible for Influenza‐Like Illness,” medRxiv January 2021:2020.02.04.20020404. 10.1016/j.jtbi.2022.111145 35490763

