# Supplementary material for: Temporal Trends in Respiratory Infection Epidemics Among Pediatric Inpatients Throughout the Course of the COVID‐19 Pandemic From 2018 to 2023 in Fukushima Prefecture, Japan
Source: Influenza Other Respir Viruses. 2025 Jan 12;19(1):e70070. doi: 10.1111/irv.70070 (PMC11725402; doi:10.1111/irv.70070)
Supplement: Supplementary file 1 — Table S1. Trends in the age of hospitalized children according to the virus from 2018 to 2023 [file IRV-19-e70070-s001.docx]

**Supplementary Table. Trends in the age of hospitalized children according to the virus from 2018 to 2023**

RSV A RSV B

| year | number | mean | median | IQR |
| --- | --- | --- | --- | --- |
| 2018 | 43 | 18.0 | 15.0 | 5.0–24.5 |
| 2019 | 77 | 25.8 | 16.0 | 3.0–30.0 |
| 2020 | 10 | 34.5 | 12.0 | 3.3–37.8 |
| 2021 | 97 | 22.5 | 17.5 | 3.0–31.0 |
| 2022 | 42 | 24.9 | 17.0 | 8.0–32.0 |
| 2023 | 38 | 24.2 | 17.0 | 7.0–35.0 |

| year | number | mean | median | IQR |
| --- | --- | --- | --- | --- |
| 2018 | 82 | 13.7 | 11.0 | 2.3–19.8 |
| 2019 | 75 | 23.4 | 14.0 | 4.5–30.0 |
| 2020 | 3 | 26.3 | 17.0 | 16.0–32.0 |
| 2021 | 9 | 22.7 | 14.0 | 2.0–18.0 |
| 2022 |  |  |  |  |
| 2023 | 65 | 26.1 | 14.0 | 4.0–32.0 |

Flu A Flu C

| year | number | mean | median | | IQR |
| --- | --- | --- | --- | --- | --- |
| 2018 | 9 | 42.9 | 26.0 | | 20.0–48.0 |
| 2019 | 15 | 62.4 | 50.0 | | 29.5–95.5 |
| 2020 | 10 | 46.3 | 45.0 | | 16.5–72.5 |
| 2021 |  |  | |  |  |
| 2022 |  |  |  | |  |
| 2023 | 15 | 70.3 | 67.0 | | 42.5–91.5 |

| year | number | mean | median | | IQR |
| --- | --- | --- | --- | --- | --- |
| 2018 | 5 | 13.0 | 12.0 | | 10.0–16.0 |
| 2019 |  |  |  | |  |
| 2020 |  |  |  | |  |
| 2021 |  |  | |  |  |
| 2022 |  |  |  | |  |
| 2023 | 16 | 43.0 | 19.0 | | 15.0–44.5 |

HCoV OC43 HCoV NL63

| year | number | mean | median | IQR |
| --- | --- | --- | --- | --- |
| 2018 | 6 | 23.0 | 20.5 | 13.3–32.3 |
| 2019 | 4 | 18.0 | 15.0 | 0.8–32.3 |
| 2020 | 8 | 32.1 | 17.0 | 10.8–28.8 |
| 2021 | 13 | 28.2 | 29.0 | 18.0–39.0 |
| 2022 |  |  |  |  |
| 2023 | 10 | 38.0 | 27.5 | 7.8–72.5 |

| year | number | mean | median | IQR |
| --- | --- | --- | --- | --- |
| 2018 | 3 | 26.0 | 16.0 | 14.5–32.5 |
| 2019 | 7 | 24.0 | 23.0 | 22.3–30.0 |
| 2020 |  |  |  |  |
| 2021 | 12 | 26.3 | 24.0 | 13.8–28.0 |
| 2022 | 1 |  |  |  |
| 2023 | 4 | 30.5 | 18.0 | 12.0–36.5 |

| year | number | mean | median | IQR |
| --- | --- | --- | --- | --- |
| 2018 | 37 | 28.5 | 24.0 | 13.0–36.0 |
| 2019 | 37 | 22.0 | 16.0 | 12.0–30.0 |
| 2020 | 23 | 25.0 | 20.0 | 13.5–44.0 |
| 2021 |  |  |  |  |
| 2022 | 22 | 27.1 | 26.0 | 17.8–38.5 |
| 2023 | 29 | 37.8 | 20.0 | 12.0–53.0 |

HCoV HKU1 　　　　　　　　　　　　　　　　　　　　　　HMPV

| year | number | mean | median | IQR |
| --- | --- | --- | --- | --- |
| 2018 | 4 | 14.5 | 11.5 | 1.0–25.0 |
| 2019 | 5 | 37.0 | 46.0 | 34.0–46.0 |
| 2020 | 6 | 65.5 | 47.0 | 30.3–80.3 |
| 2021 |  |  |  |  |
| 2022 |  |  |  |  |
| 2023 | 12 | 52.1 | 42.0 | 21.5–52.8 |

HPIV 1　　　　　　　　　　　　　　　　　　　　　　　　HPIV 2

| year | number | mean | median | IQR |
| --- | --- | --- | --- | --- |
| 2018 | 9 | 32.0 | 27.0 | 13.0–45.0 |
| 2019 | 5 | 17.4 | 12.0 | 2.0–31.0 |
| 2020 | 1 |  |  |  |
| 2021 |  |  |  |  |
| 2022 | 14 | 31.2 | 28.5 | 14.3–37.3 |
| 2023 | 7 | 31.4 | 26.0 | 14.5–50.5 |

| year | number | mean | median | IQR |
| --- | --- | --- | --- | --- |
| 2018 |  |  |  |  |
| 2019 | 5 | 51.2 | 66.0 | 12.0–74.0 |
| 2020 |  |  |  |  |
| 2021 | 1 |  |  |  |
| 2022 | 2 |  |  |  |
| 2023 | 9 | 64.7 | 69.0 | 59.0–84.0 |

HPIV 3　　　　　　　　　　　　　　　　　　　　　　　　HPIV 4

| year | number | mean | median | IQR |
| --- | --- | --- | --- | --- |
| 2018 | 28 | 14.6 | 13.0 | 10.0–19.0 |
| 2019 | 28 | 17.8 | 13.0 | 10.0–19.0 |
| 2020 | 1 |  |  |  |
| 2021 | 20 | 21.8 | 20.5 | 11.8–28.3 |
| 2022 | 1 |  |  |  |
| 2023 | 29 | 34.2 | 24.0 | 17.0–42.0 |

| year | number | mean | median | IQR |
| --- | --- | --- | --- | --- |
| 2018 | 4 | 17.8 | 16.0 | 14.0–19.8 |
| 2019 | 10 | 19.1 | 15.5 | 10.5–17.8 |
| 2020 | 1 |  |  |  |
| 2021 | 6 | 27.8 | 20.5 | 16.0–40.8 |
| 2022 | 4 | 41.0 | 42.0 | 23.5–59.5 |
| 2023 | 11 | 27.5 | 17.0 | 12.0–44.0 |

HAdV 2　　　　　　　　　　　　　　　　　　　　　　　　HAdV 4

| year | number | mean | median | IQR |
| --- | --- | --- | --- | --- |
| 2018 | 59 | 20.6 | 18.0 | 11.0–26.5 |
| 2019 | 39 | 25.1 | 17.0 | 12.0–37.0 |
| 2020 | 23 | 22.0 | 17.0 | 10.5–30.0 |
| 2021 | 23 | 28.1 | 18.5 | 14.0–29.8 |
| 2022 | 38 | 25.5 | 19.0 | 14.0–28.8 |
| 2023 | 33 | 29.0 | 22.0 | 9.0–35.0 |

| year | number | mean | median | IQR |
| --- | --- | --- | --- | --- |
| 2018 | 13 | 33.8 | 35.0 | 16.0–41.0 |
| 2019 | 26 | 31.1 | 27.5 | 10.3–47.8 |
| 2020 | 7 | 39.1 | 36.0 | 27.0–39.0 |
| 2021 | 4 | 13.8 | 11.0 | 3.5–21.3 |
| 2022 | 3 | 46.0 | 16.0 | 16.0–61.0 |
| 2023 | 6 | 24.5 | 27.5 | 8.8–41.0 |

| year | number | mean | median | IQR |
| --- | --- | --- | --- | --- |
| 2018 | 52 | 14.8 | 12.0 | 4.0–22.3 |
| 2019 | 67 | 27.1 | 13.5 | 6.0–38.8 |
| 2020 | 54 | 29.9 | 19.0 | 11.3–38.3 |
| 2021 | 49 | 25.3 | 19.0 | 12.8–30.3 |
| 2022 | 67 | 29.7 | 18.0 | 8.0–36.0 |
| 2023 | 81 | 29.1 | 21.0 | 13.0–41.0 |

HBoV　　　　　　　　　　　　　　　　　　　　　　　　HRV

| year | number | mean | median | IQR |
| --- | --- | --- | --- | --- |
| 2018 | 68 | 21.2 | 17.0 | 11.0–25.3 |
| 2019 | 50 | 21.8 | 14.5 | 12.0–29.8 |
| 2020 | 25 | 33.3 | 16.0 | 11.0–20.0 |
| 2021 | 41 | 24.6 | 17.0 | 12.0–23.0 |
| 2022 | 30 | 24.0 | 13.0 | 8.0–25.5 |
| 2023 | 22 | 32.4 | 18.5 | 12.0–38.8 |

Age is shown in months. Median and IQR for age are shown in the table for years in which the virus was detected in five or more persons.

IQR, interquartile range; RSV, respiratory syncytial virus; Flu, influenza virus; HCoV, human coronavirus; HMPV, human metapneumovirus; HPIV, human parainfluenza virus; HAdV, human adenovirus; HBoV, human bocavirus; HRV, human rhinovirus
